# Knowledge, risk perception and preparedness towards coronavirus disease-2019 (COVID-19) outbreak among Ghanaians: a quick online cross-sectional survey

**DOI:** 10.11604/pamj.supp.2020.35.2.22630

**Published:** 2020-05-20

**Authors:** Dorcas Serwaa, Emmanuel Lamptey, Anthony Baffour Appiah, Ephraim Kumi Senkyire, Jude Kyeremeh Ameyaw

**Affiliations:** 1 Institute of Life and Earth Sciences (Including Health and Agriculture), Pan African University, University of Ibadan, Ibadan, Nigeria; 2 Ghana Field Epidemiology and Laboratory Training Program (GFELTP), School of Public Health, University of Ghana-Legon, Accra, Ghana; 3 Department of Paediatric Nursing, Cape Coast Teaching Hospital, Cape Coast, Ghana

**Keywords:** COVID-19, knowledge, risk perceptions, preparedness, Ghana

## Abstract

**Introduction:**

Coronavirus disease 2019 (COVID-19) is recognized as global pandemic, affecting more than 300,000 worldwide. Ghana joined the international community by confirming first two COVID-19 cases on March 12, 2020. The study aimed to assess the public knowledge, risk perception and preparedness to respond the COVID-19 in the early stage of the outbreak in Ghana.

**Methods:**

A cross-sectional study was conducted to collect information from Ghanaian during the early stage of the outbreak from 12th to 20th March 2020. Electronic based questionnaire was developed to collected information on the public knowledge, risk perceptions and preparedness to respond the COVID-19. All people who were aged 18 years and over were invited to participate in the study.

**Results:**

A total of 350 participants were recruited into the analysis; 56% were males, with the majority of the study population aged between 18-30 years (61.4%), single (68.9%) and attained tertiary education (95.1%). Regarding COVID-19, 62.7% had “good” knowledge about the outbreak, 68.3% had a high risk of contracting the COVID-19 infection and 81.4% had a moderate preparedness skill to prevent and control the disease. Internet (77.1%) was the major sources of information. Knowledge of COVID-19 was significantly associated with education (p<0.001), age (p=0.018), employment (p=0.011) and health-related occupation (P=0.001) but only religion was associated with risk perception.

**Conclusion:**

Though overall public knowledge was good, disparity exist among the least educated population, there was high risk perceptions and moderate preparedness skill to respond to COVID-19 among our study population. We recommend that educational campaigns through timely online update on COVID-19, van mobilization and mass media broadcasting should target all groups including those in the rural communities.

## Introduction

The emergence and sporadic spread coronavirus (COVID-19) disease from Wuhan City, Hubei Province of China has become a global health concern [1-3]. The COVID-19 was identified as a novel and contagious primary atypical (viral) pneumonia reported to cause clusters of onset similar to severe acute respiratory syndrome coronavirus (SARS-CoV) and Middle East respiratory syndrome coronavirus (MERS-CoV) [4–6]. The commonest clinical features include fever, cough, acute respiratory distress, reduced or normal white blood cells, fatigue and failure to resolve over 3 to 5 days of antibiotic treatment [7]. The World Health Organization (WHO) declared the novel coronavirus disease 2019 (COVID-19) a public health emergency of international concern on January 30, 2020 [8]. In the sense that, COVID-19 outbreak was unique in terms of high pathogenicity and mortality compared to the previous epidemics by coronaviruses [2,9,10].

The pattern of sporadic spread and importation of COVID-19 in Africa affirms previously published report assessing preparedness and vulnerability of African countries against importations of COVID-19 [11]. The study identified Egypt, Algeria, and South Africa as countries with the highest importation risk and have moderate to high capacity to respond to outbreaks. On the contrary, Nigeria, Ethiopia, Sudan, Angola, Tanzania, Ghana and Kenya were classified as moderate risk and have variable capacity and high vulnerability [11]. As at April, 2nd 2020, the World Health Organization has recorded 896450 confirmed cases of COVID-19 with 45526 deaths worldwide. Limited local transmission inside China with vast importations in Africa countries [12]. World statistics shows that all continents reported confirmed cases of COVID-19 while Africa remain at the bottom. Africa confirmed its first case in Egypt on Feb 14, 2020 [11].

Thereafter, Africa has seen a sporadic increased in both importations and local transmission, 1 in Feb 14, 2020 to 4702 confirmed in April 2, 2020 [12]. Prudent measures were implemented to prevent and control importation of cases from foreign countries, however, these measures failed [13]. On March 12, 2020, Ghana recorded first two cases of COVID-19 and as at April 2, 2020, authorities have recorded 204 confirmed cases with 31 recoveries and 5 deaths. In efforts to fight against this pandemic, In efforts to fight against this pandemic, better understanding of existing measures of detection, prevention, and control among the general public cannot be underrated. The population awareness and knowledge must be heightened on containment guidelines such as surveillance and rapid identification of suspected cases, patient transfer and isolation, rapid diagnosis, tracing and follow-up of potential contacts [11]. Poor public awareness about these measures could associate with the public´s emotional and behavioral reactions towards the COVID-19.

A timely understanding of the public’s awareness, knowledge, risk perception of the COVID-19 and their influence on individuals’ and preparedness is still lacking in Ghana. Jian-Bin et al. [14] confirmed that public´s knowledge, perception, precautionary behavior and active social participation have been found to be important in the control of epidemics, as learned regarding SARS, Ebola and H1N1. Knowledge, risk perceptions and preparedness to respond the COVID-19 among the general population is essential to contain the diseases at the community level [14-16]. The present study aims to assess the knowledge, risk perceptions and preparedness to respond the coronavirus disease 2019 (COVID-19) among Ghanaians in the early phase of the outbreak. The information from the study would enable government, government spokespeople and other stakeholders such as the media and health organizations to implement adequate responses. The findings will directly improve communication measures and public education and also support policy development and public health implementation to quickly respond to the outbreak in a short and sustained manner.

## Methods

**Study design:** a cross-sectional study was conducted to collect the information on the publics´ knowledge, risk perceptions and preparedness to respond COVID-19, from March 12 -18, 2020. We adopted this snapshot method using google forms via electronic platforms in order to meet the objective of the study, limit physical contact with respondents and get responses quickly as far as possible during the early period of outbreak.

Sample size estimation: we estimated the minimum sample size using the Cochran formula [17] and based on the information from a previous study; given that Z (at 95% confidence interval)=1.96, p (proportion with good knowledge)=0.27, q (1-p)= 0.73 and e (margin of error) =0.05, 303 participants were required. Factoring 10% non-response rate during the study, at least 350 participants were needed for the analysis.

**Data collection:** we adopted and modified survey questionnaires from previous studies on similar subject about COVID-19 [18]. The survey questionnaires assessed the relevant sociodemographic characteristic of respondents, knowledge, risk perception and preparedness midst the outbreak. The survey questionnaires were shared on all electronic platforms and online social media like facebook, emails, WhatsApp, instagram, twitter etc. to groups, tertiary students, friends, family members and civil servants. These accessible populations also shared and forwarded the survey to their contacts and various groups to keep the survey widely distributed as far as possible. The questionnaire was made of four sections, with each section assessing respectively the demographic characteristic, knowledge, risk perception and preventive measures of individual respondent midst the outbreak.

The first section elicited information regarding socio-demographic background of the respondents´ (gender, age, religion, marital status, level of education, employment etc.). In the second section, 12 questions were used to collect information regarding knowledge related to COVID-19. Each answer was graded from 0 (incorrect answers) and 1 (correct answers). The maximum score a respondent could obtain was be 12 and a minimum of 0. Based on the scores, 0-5 were classified as having “poor” knowledge, scores of 6-9 were classified as “moderate” knowledge and scores of 10-12 were classified as “good” knowledge. In part three of the questionnaire, three questions with three responses options each were asked to determine the respondents risk perception. Each answer was graded from 0 (disagree) to 2 (agree). The maximum score patients could obtain will be 6, the minimum was 0.

People who scored 0-2 were classified as having low-risk perception, a score of 3-4 were classified as having moderate-risk perception and high-risk perception were scored 5-6. Section four contained 5 questions regarding preparedness to respond to COVID-19 in the area, such as surgical mask use, hand washing, using soap to wash the hands, using sanitizer and avoidance of face touching. Three response options were provided for each item: every time, sometimes and never. The maximum score a respondent could obtain was be 10 and a minimum of 0. Scores between 0-3 indicated a poor level of preparedness to respond to the outbreak of COVID-19, scores of 4-6 indicated a moderate level of preparedness and scores of 7-10 indicated a good level of preparedness.

**Data analysis:** data collected were extracted using Excel version 2016 and imported into IBM SPSS version 22 for analysis. We analyzed data descriptively using frequency and proportions. A chi-square test (to analyze the factors influencing knowledge and preventive measures of the novel coronavirus) was computed. The confidence level was 95% and statistical significance set at p<0.05.

**Ethical consideration:** we followed strictly “Declaration of Helsinki-Ethical Principles for Medical Research” throughout the study. Participants aged below 18 years were excluded because of their vulnerability as minors. Prior to participation, the purpose of study, the confidential and voluntary nature of the survey and the estimated time it will take to complete the questionnaire were explained to potential respondents. Respondents were also informed that by choosing to access the survey link, they are providing their consent to participate.

## Results

**Background of respondents:** Table 1 depicts the main sociodemographic characteristics of the study participants. A total of 350 respondents participated in this study; 196/350 (56%) were males. The age varied between 18-60 years and most encountered group 215/350 (61.4%) was within 18-30 years. Almost all were Christians (85.7%), single (68.9%) and have attained tertiary education (95.1%). Majority 228/350 (65.1%) were employed, 124/350 (54.4) worked in health-related institutions and 17.1% of the work involved travelling and or crowd-related.

Knowledge of respondents about COVID-19: with regard to knowledge on COVID-19, almost all the participants knew it was viral infection (96.6%) and originated from China (99.4%). Nearly 73% of the respondents cited correctly the incubation period of the virus as between 2-14 days. Most (47.3%) of the participants cited that the disease was a treatable. Two hundred and forty-eight participants (248/350, 70.9%) had a correct understanding of the non-availability of a vaccine for COVID-19. Fever (95.1%), cough (97.4%), difficulty in breathing (92.3%) and sore throat (77.7%) were the most citied signs and symptoms of the disease. The majority (77.1%) received information through internet, 46.9% reported that the information received was not sufficient for making decisions. Half of the respondents (50.9%) trusted the source of information and the majority (72.9%) knew Ghana´s COVID-19 emergency contact and facility (Table 2).

**Table 1:** Socio-demographic characteristics of the study participants

Variable	number (n)	Percentage (%)
**Gender**		
Male	196	56.0
Female	154	44.0
**Age**		
18-30	215	61.4
31-45	122	34.9
46-60	13	3.7
**Religion**		
Christian	300	85.7
Muslim	42	12.0
Others	8	2.3
**Marital status**		
Single	241	68.9
Married	106	30.3
Ever married	3	0.9
**Education**		
High School	17	4.9
Tertiary	333	95.1
**Employment**		
Employed	228	65.1
Unemployed	122	34.9
**Type of Occupation**		
Health-related	124	54.4
Not health-related	110	45.6
**Travelling or crowd related Occupation**		
Yes	39	17.1
No	194	82.9

**Table 2:** Participants’ knowledge, source of information, risk perception and preparedness of coronavirus

Variable	(n)	(%)
Knowledge about the coronavirus (*) COVID- 19		
Viral infection	338	96.6
Bacterial infection	8	2.3
Protozoan infection	1	0.3
Not Sure	3	0.9
**COVID-19 origin**		
China	348	99.4
Africa	2	0.6
**How many days does it take for infected person to show symptoms**		
Immediately one is exposed	90	25.7
between two and 14 days	255	72.9
between 14 and 30 days	3	0.9
not sure	2	0.6
**Treatment for the COVID-19 disease**		
Yes	63	18.0
No	213	60.9
Not sure	74	21.1
**Availability of a vaccine for COVID-19**		
Yes	28	8.0
No	248	70.9
Not sure	74	21.1
**Major signs and symptoms of COVID-19 infection**		
Fever	333	95.1
Cough	341	97.4
difficulty breathing	323	92.3
muscle pain or tiredness	139	39.7
Sore throat	272	77.7
Running nose	229	65.4
Diarrhea and Blurred vision	51	14.6
**Information on COVID-19**		
**Main channel of receiving COVID-19 information**		
Internet	270	77.1
Television	177	50.6
Medical staff	132	37.7
Friends and Relatives	147	42.0
**Sufficient Information available on COVID-19**		
Yes	152	43.4
No	164	46.9
Not Sure	34	9.7

**Respondent´s risk perception attitude:** regarding perception of risk, majority (68.3%) of the study participants had high-risk perception towards the COVID-19. Regarding the perception of the dangerousness of COVID-19, 84.9% considered the disease dangerous. A total of 78.3% reported feeling worried about the outbreak and 67.4% reported that there was a high risk of contracting the COVID-19 infection. About half of the people correctly stated that the high-risk group of COVID-19 infection are old aged people (Table 2 (suite)).

**Table 2 (suite):** Participants’ knowledge, source of information, risk perception and preparedness of coronavirus

Level of trust regarding sources of information	(n)	(%)
Strong	178	50.9
Neutral	140	40.0
Weak	32	9.1
**Aware of any COVID-19 emergency contact or facility**		
Yes	255	72.9
**No**		
Risk Perception	95	27.1
**Dangerousness of COVID-19**		
Dangerous	297	84.9
Like the common flu	47	13.4
Not dangerous	6	1.7
**Worrying about COVID-19**		
Worried	274	78.3
Worried about it as if it were the common flu	43	12.3
Not worried	33	9.4
**Level of risk of contracting COVID-19 infection**		
High risk	236	67.4
Risk similar to that of contracting the common cold	111	31.7
No risk	3	0.9
**Group of people more at risk**		
Children	19	5.4
Old age people	191	54.6
Young adults	9	2.6
All people	127	36.3
**Not sure**		
Feeling of preparedness to avoid an infection with the coronavirus	4	1.1
**Surgical mask use**		
Every day at all times	113	32.3
Sometimes	153	43.7
Never	84	24.0
**Frequency of washing hands per day**		
More than 3 times a day	320	91.4
1-3 times	30	8.6
**Using soap to wash hands**		
Every time	313	89.4
Sometimes	37	10.6
**Using sanitizers to disinfect the hands**		
Every time	253	72.3
Sometimes	88	25.1
Never	9	2.6
**Touching of Face**		
Every time	21	6.0
Sometimes	102	29.1
Never	227	64.9

**Preparedness skills of respondents:** regarding the feeling of preparedness to avoid an infection with the coronavirus, minority (32.3%) of the respondents regularly used a surgical mask, 91.4% washed their hands more than 3 times per day, 89.4% did regularly use soap while washing their hands, 72.3% used sanitizers to disinfect their hands every time and only 6.0% regularly touched their faces (Table 2 (suite)).

**Figure 1:**
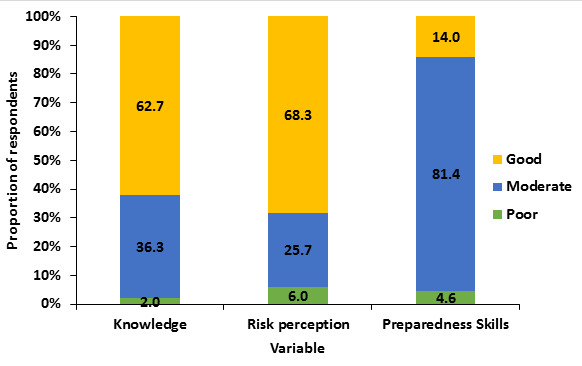
Comparison of overall knowledge, risk perception and preparedness skills among respondents

**The overall Knowledge, perception and preparedness skills:** generally, 216/350 (61.7%) of the respondents had “good” knowledge about the COVID-19 outbreak, 68.3% had high risk perception and the majority (81.4%) of the study participants had a moderate preparedness skill (Figure 1).

**Bivariate analysis:** a chi-square test was adopted in analyzing the factors influencing knowledge, risk perception and preparedness the novel coronavirus at 95% confidence level and statistical significance set at p<0.05. With regard to knowledge about the COVID-19, the chi-square test revealed that age (p=0.018), marital status (p=0.006), education (p<0.001), employment (p=0.011), type of occupation (p=0.001), sufficiency of available information (p=0.007) and the main channel of receiving information (p=0.022) and awareness of COVID-19 emergency contact number or facility (p=0.050) were the factors that significantly influenced the level of knowledge of the respondents. Religion was the only factor that significantly influenced the risk perception attitude of the respondents (p=0.001). The respondents feeling of preparedness towards this outbreak was significantly linked to their levels of education (p=0.001), type of occupation (p=0.001) and the main channel of receiving information (p=0.006) (Table 3, Table 3 (suite) and Table 3 (suite 1)).*Check end of document

**Table 3:** Comparison of socio-demographic characteristics with knowledge, risk perception and preparedness

Characteristics	Knowledge				Risk perception				Preparedness			
	Poor N (%)	Moderate N (%)	Good N (%)	χ2 (p-value)	Low N (%)	Moderate N (%)	High N (%)	χ2 (p-value)	Poor N (%)	Moderate N (%)	Good N (%)	χ2 (p-value)
**Gender**												
Male	2 (1.0)	73 (37.2)	121 (61.7)	2.250 (0.36 a)	13 (6.63)	59 (30.10)	124 (63.27)	5.277 (0.071)	7 (3.57)	161 (82.14)	28 (14.29)	1.028 (0.598)
Female	5 (3.2)	54 (35.1)	95 (61.7)		8 (5.19)	31 (20.13)	115 (74.68)		9 (5.84)	124 (80.52)	21 (13.64)	
**Age**												
18-30	7 (3.26)	88 (40.93)	120 (55.81)	11.430 (0.018*, a)	18 (8.37)	51 (23.72)	146 (67.91)	6.951 (0.075 a)	9 (4.19)	179 (83.26)	27 (12.56)	4.100 (0.279 a)
31-45	0 (0.00)	34 (38.46)	88 (72.54)		2 (1.62)	35 (28.69)	85 (69.67)		6 (4.69)	98 (80.33)	18 (14.75)	
46-60	0 (0.00)	5 (36.29)	8 (61.54)		1 (7.69)	4 (30.77)	8 (61.54)		1 (7.69)	8 (61.54)	4 (30.77)	
**Religion**												
Christian	5 (1.67)	111 (37.00)	184 (61.33)	12.215 (0.372 a)	18 (6.00)	81 (27.00)	201 (67.00)	22.644 (0.001*, a)	13 (4.33)	245 (81.67)	42 (14.00)	1.106 (0.860 a)
Muslim	2 (4.76)	12 (28.57)	28 (66.67)		0 (0.00)	6 (14.29)	36 (85.71)		3 (7.14)	33 (78.57)	6 (14.29)	
Others	0 (0.00)	4 (50.0)	4 (50.0)		3 (37.50)	3 (37.50)	2 (25.00)		0 (0.00)	7 (87.50)	1 (12.50)	

* Significance level at α=0.05, aFisher’s exact test

**Table 3 (suite):** Comparison of socio-demographic characteristics with knowledge, risk perception and preparedness

Characteristics	Knowledge				Risk perception				Preparedness			
	Poor N (%)	Moderate N (%)	Good N (%)	χ2 (p-value)	Low N (%)	Moderate N (%)	High N (%)	χ2 (p-value)	Poor N (%)	Moderate N (%)	Good N (%)	χ2 (p-value)
**Marital status**												
Single	7 (2.00)	99 (36.29)	216 (61.71)	12.215 (0.006*, a)	20 (8.30)	61 (25.31)	160 (66.39)	7.345 (0.052 a)	11 (4.46)	200 (82.99)	30 (12.45)	2.310 (0.475 a)
Married	0 (0.00)	27 (25.47)	79 (74.53)		1 (0.94)	28 (26.42)	77 (72.64)		5 (4.72)	83 (78.30)	18 (16.98)	
Ever married	0 (0.00)	1 (33.33)	2 (66.67)		0 (0.00)	1 (33.33)	2 (66.67)		0 (0.00)	2 (66.67)	1 (33.33)	
**Education**												
High School	2 (11.76)	12 (70.59)	3 (17.65)	19.935 (<0.001*, a)	1 (5.88)	2 (11.76)	14 (82.35)	1.870 (0.372 a)	0 (0.00)	10 (58.82)	7 (41.18)	11.364 (0.012*, a)
Tertiary	5 (1.50)	115 (34.53)	213 (63.96)		20 (6.01)	88 (26.43)	225 (67.57)		16 (4.80)	275 (82.58)	42 (12.61)	
**Employment**												
Employed	3 (1.32)	72 (31.58)	153 (67.11)	8.6048 (0.011*, a)	12 (5.26)	57 (25.00)	159 (69.74)	0.923 (0.630)	10 (4.39)	185 (81.14)	33 (14.47)	0.161 (0.923)
Unemployed	4 (3.28)	55 (45.08)	63 (61.71)		9 (7.38)	33 (27.05)	80 (65.57)		6 (4.92)	100 (81.43)	16 (13.11)	
**Type of Occupation**												
Health-related	1 (0.81)	28 (22.58)	95 (76.61)	11.761 (0.001*, a)	8 (6.45)	29 (23.39)	87 (70.16)	1.457 (0.483)	1 (0.81)	110 (88.71)	13 (10.48)	13.542 (0.001)
Not health-related	2 (1.28)	47 (42.73)	61 (55.45)		4 (3.64)	31 (28.18)	75 (68.18)		10 (9.09)	79 (71.82)	21 (14.53)	

**Table 3 (suite 1):** Comparison of socio-demographic characteristics with knowledge, risk perception and preparedness

	Knowledge				Risk perception				Preparedness			
Travelling or crowd related	Poor N (%)	Moderate N (%)	Good N (%)	χ2 (p-value)	Low N (%)	Moderate N (%)	High N (%)	χ2 (p-value)	Poor N (%)	Moderate N (%)	Good N (%)	χ2 (p-value)
Yes	3 (1.55)	58 (31.76)	133 (68.56)	2.314 (0.306 a)	8 (5.15)	49 (25.26)	137 (70.62)	2.868 (0.205 a)	10 (5.15)	154 (79.38)	30 (15.46)	2.276 (0.428 a)
No	0 (0.00)	16 (41.03)	23 (58.97)		4 (10.26)	11 (28.21)	24 (61.54)		1 (2.56)	35 (89.74)	3 (7.69)	
**Main channel of receiving 2019 novel coronavirus information**												
Internet	5 (1.90)	92 (34.00)	173 (64.10)	17.957 (0.022*, a)	16 (5.90)	68 (25.2)	186 (68.9)	10.117 (0.257)	11 (4.10)	229 (84.80)	30 (11.10)	
Television	1 (0.60)	60 (33.90)	116 (65.50)		9 (5.1)	40 (22.6)	128 (72.3)		9 (5.10)	151 (85.30)	17 (9.60)	21.506 (0.006*)
Medical staff	0 (0.00)	41 (31.10)	91 (61.20)		6 (4.50)	38 (28.80)	88 (66.7)		3 (2.30)	113 (85.60)	16 (12.10)	
Friends and Relatives	1 (0.07)	56 (38.10)	90 (61.20)		14 (9.50)	37 (25.20)	96 (65.30)		6 (4.10)	115 (78.20)	26 (17.70)	
**Sufficient Information available on COVID-19**												
Yes	1 (2.00)	127 (36.30)	96 (63.2)	13.03 (0.007*, a)	11 (6.00)	41 (27.00)	100 (65.80)	1.488 (0.832 a)	7 (4.60)	126 (82.90)	19 (12.5)	0.946 (0.931a)
No	2 (1.20)	57 (34.80)	105 (64.0)		8 (4.90)	42 (25.60)	114 (65.50)		8 (4.90)	132 (80.50)	24 (14.6)	
Not Sure	4 (11.8)	15 (44.10)	15 (44.10)		2 (5.90)	7 (20.60)	25 (73.50)		1 (2.90)	126 (82.90)	19 (12.5)	
**Aware of any COVID-19 emergency contact or facility**												
Yes	3 (1.20)	87 (34.10)	165 (64.70)	5.643 (0.050*, a)	18 (7.10)	70 (27.50)	167 (65.50)	3.933 (0.142)	8 (3.10)	212 (83.10)	35 (13.7)	4.416 (0.104a)
No	4 (4.20)	40 (42.1)	51 (53.7)		3 (3.30)	20 (21.10)	72 (75.80)		8 (8.40)	73 (76.80)	14 (14.7)	

* Significance level at α=0.05, aFisher’s exact test

## Discussion

Coronavirus disease 2019 (COVID-19) is recognized as global pandemic [19]. Ghana joined the international community by confirming first two COVID-19 cases on March 12, 2020 [3]. The number of cases has consistently increased alongside local transmission in the early stage of COVID-19 outbreak in Ghana and this has created public fear and panic among the general population. The Greater Accra leads the sixteen regions in the country, with huge inflow of people from international communities through air and land ports, making its inhabitants more vulnerable population for COVID-19 infection [13,18]. This study aimed at providing early evidence about the knowledge, risk perceptions and preparedness towards COVID-19 among Ghanaians. A greater proportion of study participants were males, aged between 18-60 years, well-educated population. Our finding showed that the overall knowledge about the COVID-19 outbreak was 61.7%. A study carried out by [20] to evaluate the knowledge and perceptions of COVID-19 among healthcare workers in United Arab Emirates found insufficient knowledge among the study population.

However, similar study conducted by Zhong et al., [21] to assess knowledge, attitudes and practices among Chinese residents towards COVID-19, recorded overall rate of 90% on the knowledge questionnaire. We unexpected to record average level of knowledge of COVID-19 among our study population. The reason being that, there exist an overwhelming news reports on this public health emergency, we assumed the population would have actively learnt knowledge of this infectious disease from various channels of information. Also, by considering the sample characteristics: 95.1% of the study respondents held at HND, diploma or higher degree, we expected them to be well informed. Despite the average level of knowledge rec orded, most of the participants knew COVID-19 is viral infection (96.6%) and originated from China (99.4%), identifying fever (95.1%), cough (97.4%) and difficulty in breathing (92.3%) as the most common symptoms of COVID-19. In addition, many of the them had accurate knowledge that COVID-19 has no first line treatment and there is a vaccine available.

Our study further revealed that the knowledge of COVID-19 was strongly associated with level of participants´ education (p<0.001), age (p=0.018), employment (p=0.011) and health-related occupation (P=0.001). People who are educated are more likely to read from several online articles, newsletters and other viral information on COVID-19 outbreak. The risk is that the vast diversity of information available through the internet, including unverified malicious information, can spread quickly and can misguide the public. As it has been revealed that widespread misinformation about COVID-19 is a serious concern causing xenophobia worldwide [20]. Also, the majority of our population who are least educated have poor knowledge of COVID-19 outbreak. Stressing our mass media, the television and local radio stations need to improve on delivery to bridge the knowledge gap between the well-educated and least educated groups. An accurate source of information on the emerging COVID-19 is essential in the combat of this public health problem.

However, a finding of considerable concern is that, the majority (77.1%) received information through internet (social media), as similarly reported by [20]. Presently, there exist vast array of information including unverified malicious information on social media and these can spread quickly and can misinform many people. Generally, most participants had a high-risk perception towards the COVID-19. About 85% of our study population perceived COVID-19 as dangerous disease, 78.3% were worried about it while 67.4% indicated they were at a high risk of contracting the COVID-19 infection. With the death tolls rising and boarders being shut down due to COVID-19 pandemic, most Ghanaians are living in fear and panic, therefore this observation was expected. About half of the people correctly stated the aged were the high-risk group of COVID-19 infection. Likewise, [3] also reported that the most vulnerable population for COVID-19 infection was older males. Also, a study in Wuhan, China, confirmed that older males with underlying diseases, such as hypertension (HT) and cardiovascular disease (CVD), were much more likely to develop infection than other groups [4].

Although, the overall risk perception was evenly distributed among various groups studied, risk perception was found to be higher among Muslims (85.7%) followed by Christians (67%). However, possible reasons why muslims perceive the highest risk of COVID-19 is not known. Our study identified religious affiliation as the only participants´ variable significantly associated with risk perception (p=0.001). Regarding public preparedness to avoid being infected with COVID-19, only one-third of the respondents (32.3%) regularly used a surgical mask, 91.4% washed their hands more than 3 times per day, 89.4% did regularly use soap while washing their hands, 72.3% used sanitizers to disinfect their hands every time and only 6.0 % regularly touched their faces during the rapid rise period of the COVID-19 outbreak. This level of preparedness could be the result of the residents´ good knowledge regarding the high infectivity of the COVID-19 virus, which can be easily transmitted between people via invisible respiratory droplets. Unfortunately, the present study still showed that 28% never wore mask when leaving homes and 2.6% did not carry hand sanitizer.

The people are probably reluctant because of the fewer number of cases confirmed in Ghana. Our study significantly linked the respondents´ feeling of preparedness towards this COVID-19 outbreak to their levels of education (p=0.001), type of occupation (p=0.001) and the main channel of receiving information (p=0.006). This implies high level of educated, been health worker and a good source of information are more likely to improve the preparedness level and adherence to COVID-19 preventive and control guidelines among Ghanaians. Findings suggest that health authorities should make extra effort to communicate existing COVID-19 preventive measures to Ghanaians as recommended by WHO [19]. Timely dissemination of information about preventive measure will not only promote public awareness but rather improve attitude and cooperation among the general public.

Strength and limitations: the developed questionnaire was close-ended and pilot tested to reduce information bias. This is a cross-sectional study conducted online among Ghanaians during the early stage of the COVID-19 outbreak. Also, to the best of our knowledge, no data have been published on the knowledge, risk perceptions and preparedness among people who are living in Ghana and Africa at large. However, the study was not without some limitations. First, due to limited access to the internet and online health information resources, vulnerable populations of Ghanaian society and illiterates were not captured in the study. Future studies should therefore target the vulnerable and illiterate populations. In addition, the data presented in this study are self-reported and partly dependent on the participants’ honesty and recall ability; thus, they may be subject to recall bias. Irrespective of these limitations, our findings provide valuable information about the knowledge, risk perception and preparedness of Ghanaians during this period of COVID-19 pandemic.

## Conclusion

Ghana has joined the international world in fight against COVID-19 pandemic. For that matter, early evidence to the understanding of public knowledge, risk perceptions and preparedness to respond the COVID-19 among Ghanaians is essential. Though overall knowledge levels were good, the least educated population are left behind, there was high risk perceptions of and moderate preparedness skill to respond to COVID-19 among our study population. As COVID-19 infection in Ghana is on ascendancy, greater efforts through educational campaigns that target all groups through timely online update on COVID-19, van mobilization in rural communities and mass media broadcasting are urgently needed.

### What is known about this topic

- Coronavirus disease 2019 (COVID-19) is recognized as global pandemic;
- Covid-19 outbreak was unique in terms of high pathogenicity and mortality compared to the previous epidemics by coronaviruses;
- No data exist on public knowledge, risk perception, preventive behavior and preparedness to respond the COVID-19 in Ghana and West Africa.

### What this study adds

- Knowledge of COVID-19 good among well-educated population with internet as main source of information;
- There was high risk perception and a moderate preparedness skill to prevent and control COVID-19;
- Findings indicate the need for health authorities to timely and effectively disseminate COVID-19 prevention and control measures to general public.

## Competing interests

The author declares no competing interests.
